# Vortioxetine as an alternative treatment for somatic symptom disorder: case report

**DOI:** 10.3389/fpsyt.2024.1496072

**Published:** 2024-11-07

**Authors:** Naoki Furutani, Yasuhide Nagoshi

**Affiliations:** ^1^ Department of Psychiatry and Neurobiology, Graduate School of Medical Science, Kanazawa University, Kanazawa, Japan; ^2^ Department of Psychiatry, Noto General Hospital, Nanao, Japan; ^3^ Department of Psychiatry (Psychosomatic Medicine), Japanese Red Cross Kyoto Daiichi Hospital, Kyoto, Japan

**Keywords:** somatic symptom disorder (SSD), anxiety disorders, obsessive-compulsive spectrum disorders (OCSD), pain, vortioxetine (VOR), serotonin reuptake inhibitor (SRI), serotonin (5-HT) receptor, case report

## Abstract

Somatic symptom disorder (SSD) is characterized by persistent physical symptoms that cause significant distress and functional impairment. Despite the widespread use of serotonin reuptake inhibitors (SRIs) in treating SSD, some patients experience insufficient response, necessitating alternative therapeutic approaches. We report two cases of SSD that demonstrated significant improvement with vortioxetine, a novel antidepressant with multimodal serotonergic receptor activity. In Case 1, an 88-year-old female with throat discomfort and cough experienced an insufficient response to an SRI. After switching to vortioxetine, she achieved significant symptom relief within 10 days, with no relapse observed over the following four months. In Case 2, a 29-year-old female presenting with widespread somatic pain and palpitations, unresponsive to analgesics, achieved symptom resolution within two weeks with the initial use of vortioxetine. The therapeutic effects of vortioxetine were rapid and well-tolerated. These cases highlight the potential of vortioxetine for treating SSD, particularly in cases of insufficient response to SRIs, and suggest a possible overlap between SSD and obsessive-compulsive spectrum disorders through its action on serotonergic pathways.

## 1 Introduction

Somatic symptom disorder (SSD) is a psychiatric disorder characterized by multiple physical symptoms that cause significant subjective distress and impair daily functioning. Its treatment is frequently challenging, especially in patients where standard antidepressants such as serotonin reuptake inhibitors (SRIs) fail to achieve adequate therapeutic outcomes. In such situations, treatment options become limited, highlighting the necessity of identifying alternative therapeutic strategies (1).

Vortioxetine (VOR) is a novel antidepressant, approved and marketed in Japan in 2019, with a hybrid mechanism, combining serotonin (5-HT) reuptake inhibition with modulation of multiple serotonin receptors, providing both antidepressant and anxiolytic effects. Notably, its agonism on 5-HT1A and antagonism on 5-HT3, 5-HT7 and 5-HT1B/1D (2–7) receptors has been shown to enhance the effects of SRIs on 5-HT pathways in preclinical studies.

In this report, we present two cases of SSD, diagnosed using the DSM-5 criteria (8), that responded favorably to VOR treatment. The first case is an 88-year-old woman whose physical symptoms, including throat discomfort and cough, had not improved with a selective serotonin reuptake inhibitor (SSRI) but showed significant relief after switching to VOR. The second case is a 29-year-old woman with widespread somatic pain and palpitations that were unresponsive to analgesics, but her symptoms resolved rapidly following the initiation of VOR. Additionally, we discuss the potential relationship between SSD and obsessive-compulsive spectrum disorders (OCSD), exploring the potential of VOR for treating these related disorders as well.

## 2 Case 1

When the patient first visited our department at the age of 78, she presented with complaints of dyspnea, palpitations, and fatigue. She had an anxious and nervous personality. After her marriage, she became a homemaker, and later, following her husband’s admission to a care facility due to dementia, she began living alone. She had a history of bilateral osteoarthritis of the knees, and over the past month, her knee pain had worsened, which caused her to feel increasingly anxious about her future. This anxiety was accompanied by discomfort in her pharynx and larynx, as well as dyspnea, palpitations, and fatigue. She became worried about her physical symptoms and visited several internal medicine clinics, but no abnormalities were found, so she consulted our hospital for further evaluation and treatment. She did not exhibit depressed mood, diminished interest or pleasure, and therefore did not meet the criteria for a depressive episode. Based on these symptoms, she was diagnosed with moderate SSD according to DSM-5 criteria (8), fulfilling Criterion B1 for disproportionate and persistent thoughts about the seriousness of her symptoms, and Criterion B2 for a persistently high level of anxiety about her health and symptoms.

Having been offered both pharmacotherapy and psychotherapy, she chose pharmacotherapy. She was initially treated with sertraline (SER), titrated up to 100 mg, the maximum dose in Japan. Although her dyspnea, palpitations, fatigue, and anxiety improved around this time, her throat discomfort persisted. The sensation worsened when she focused on it, and she began coughing repeatedly in an attempt to relieve the discomfort. Despite visiting several internal medicine and otorhinolaryngology clinics, no abnormalities were found.

Ten years later, at the age of 88, the patient began to suspect that her throat discomfort might be psychosomatic in nature and mentioned this symptom for the first time during a follow-up visit to our psychiatric department, where she had been receiving ongoing care. It was considered to be associated with her SSD. Her score on the Patient Health Questionnaire-15 (PHQ-15) (9), a common tool for assessing SSD, was 2 points around this time. Since her anxiety had decreased, and only Criterion B3 (excessive time and energy devoted to these symptoms or health concerns) was met, the severity was diagnosed as mild according to the DSM-5. However, the residual symptoms, particularly the frequent coughing that consumed a considerable amount of time, were still causing significant distress and interfering with her daily life. She expressed a desire for further pharmacotherapy to address the persistent symptoms, so her treatment was switched from SER 100 mg to escitalopram (ESC) 20 mg. However, her symptoms did not improve (PHQ-15: 2), and excessive drowsiness emerged as a side effect of ESC.

**Figure 1:**
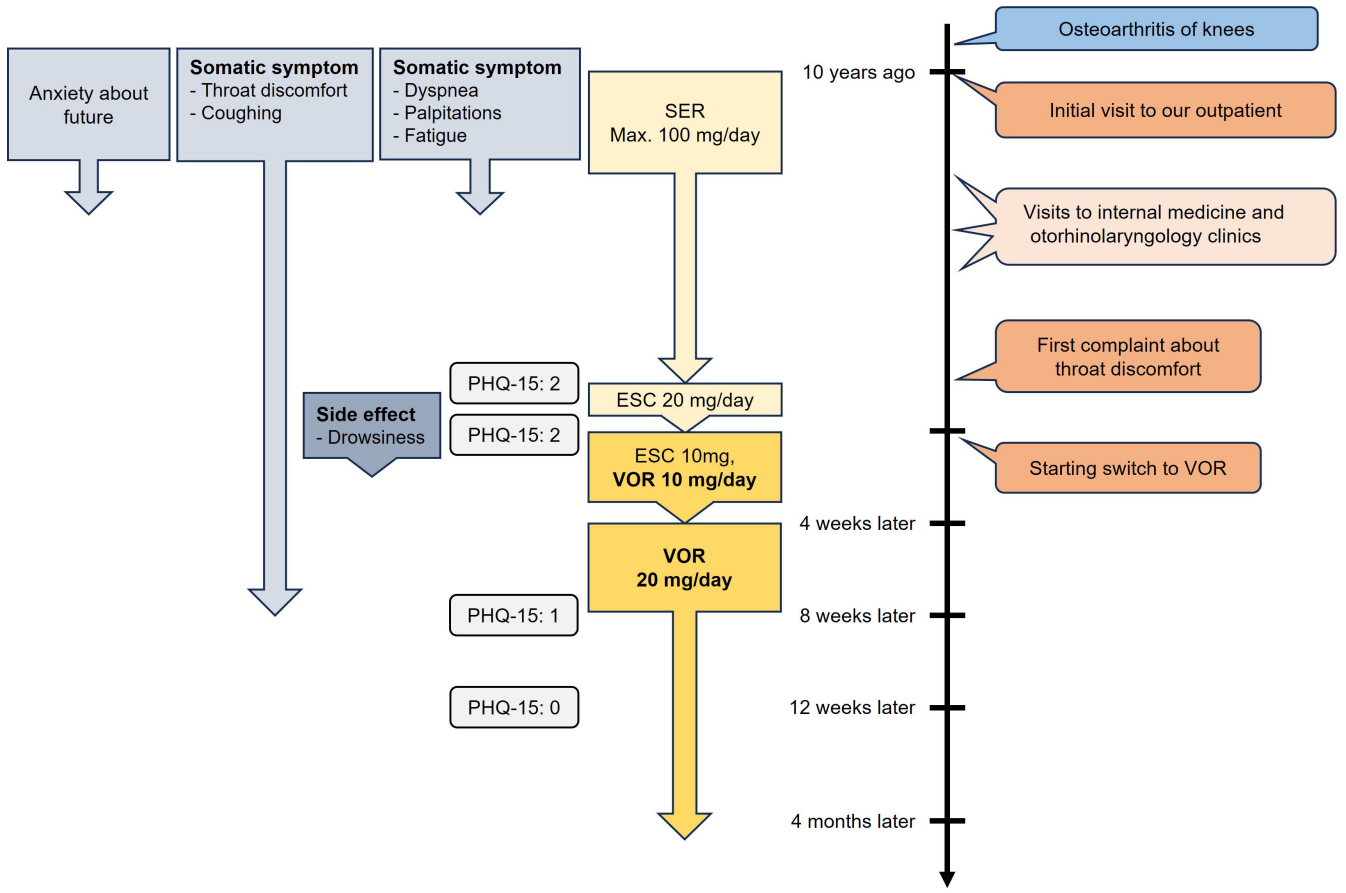
Time course of symptoms and treatments of Case 1. SER, sertraline; ESC, escitalopram; VOR, vortioxetine; PHQ-15, Patient Health Questionnaire-15.

Therefore, the treatment plan was revised to switch from ESC to VOR. ESC was tapered to 10 mg, and VOR was initiated at 10 mg. This reduced her drowsiness, but there was no significant improvement in her symptoms after four weeks. Consequently, ESC was discontinued, and VOR was titrated up to 20 mg. Monotherapy with VOR 20 mg led to the complete resolution of her drowsiness and significant improvement in her throat discomfort and coughing, which began 10 days after the dose increase and nearly disappeared after four weeks (PHQ-15: 1). Additionally, four weeks later (i.e., 12 weeks after initiating VOR), her PHQ-15 score further decreased to 0. She has now been maintained on VOR 20 mg for four months without symptom relapse, and her PHQ-15 score remains 0. Throughout this period, no adverse effects from VOR were observed, and no other psychotropic medications were administered. The patient remarked, “I think I had been paying too much attention to my throat until recently. Occasionally, I still cough, but it subsides quickly. I was lucky to talk to you about my persistent symptom that I had for many years, and it got resolved.” The time course of symptoms and treatments is summarized in **Figure 1**.

## 3 Case 2

The next case involves a 29-year-old female medical clerk who presented with symptoms of pain and discomfort in various parts of her body. Approximately one year earlier, she returned to work after maternity leave and was reassigned to a different department. Concerned about taking time off when her child was sick due to understaffing and interpersonal tensions in her workplace, she felt significant pressure and stress from not wanting to burden her colleagues. She consulted her supervisor, but the situation remained unresolved, which exacerbated her stress. Approximately six months before her initial visit to our department, she developed pain primarily around the ribs and between the shoulder blades, although the specific location of the pain varied daily. The pain became so severe that it interfered with her ability to perform housework. She was prescribed diclofenac sodium for pain management at another hospital, but her pain did not improve. Subsequently, she visited the internal medicine department of our hospital, where chest X-rays, an electrocardiogram, and blood tests, including those for thyroid function, revealed no abnormalities. She was then referred to our psychiatric department.

**Figure 2:**
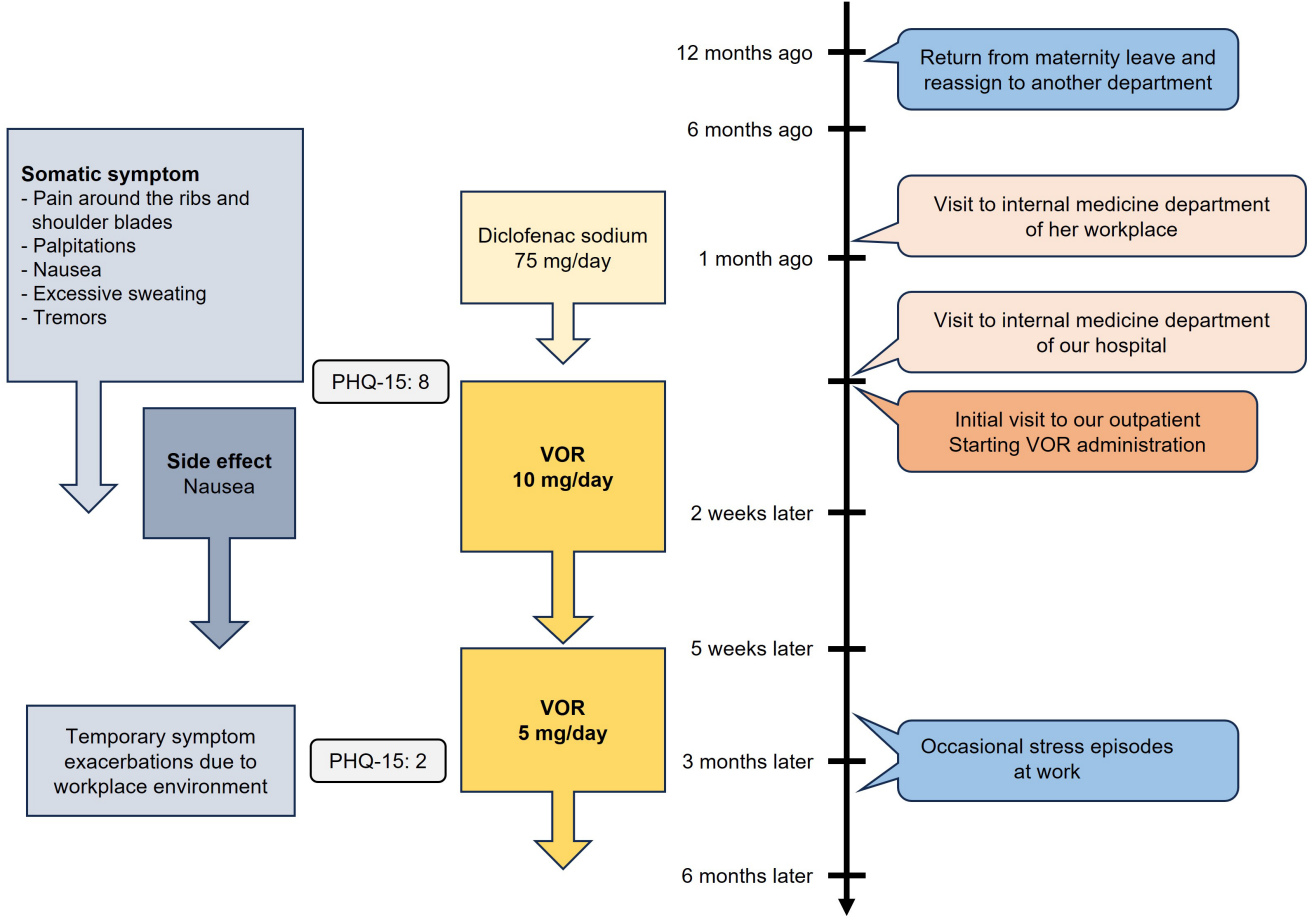
Time course of symptoms and treatments of Case 2. VOR, vortioxetine; PHQ-15, Patient Health Questionnaire-15.

The patient reported mild episodes of depressed mood or diminished interest and pleasure, each lasting less than a day, which appeared to be secondary to her primary symptom of bodily pain and did not meet the criteria for a depressive episode. She had also experienced palpitations, nausea, excessive sweating, and tremors. The patient became preoccupied with her symptoms and provided excessive detail during visits to both the internal medicine department at her workplace and at our hospital. At her initial visit, her PHQ-15 score was 8. She was diagnosed with moderate SSD based on the DSM-5 diagnostic criteria (8). The patient exhibited disproportionate and persistent thoughts about the seriousness of her symptoms (criterion B1) as well as excessive time and energy devoted to these symptoms or health concerns (criterion B3), particularly concerning her pain. Both pharmacotherapy and psychotherapy were proposed, but the patient opted for pharmacotherapy due to the practical challenges of managing household responsibilities and child-rearing. VOR was initiated at 10 mg, leading to significant improvement. VOR was selected because the patient was concerned about the potential side effect of nausea from SSRIs. Within two weeks, most symptoms, including pain, had remitted. Due to nausea as a side effect, the dose was reduced to 5 mg five weeks after starting VOR, which alleviated the side effect and improved her tolerance. The patient reported, “The pain has almost disappeared, and I now only occasionally experience palpitations. Since I no longer worry about the pain, I have been able to resume household tasks.” Thereafter, when coworkers were absent and her workload increased, she occasionally experienced diarrhea and took extended leave from work, but the symptoms did not persist, and there was no recurrence of bodily pain or preoccupation with physical symptoms. Throughout the treatment course, VOR was the only psychotropic medication administered, with no combination of other psychotropic drugs. Three months after the initial visit, her PHQ-15 score had dropped to 2. She continued taking VOR for six months before discontinuing outpatient visits on her own decision. The time course of symptoms and treatments is summarized in **Figure 2**.

## 4 Discussion

This is the first report to demonstrate the effectiveness of VOR for SSD. In the present cases, VOR showed significant efficacy in treating patients with SSD. Particularly in Case 1, the patient had shown a limited response to an SRI but improved after switching to VOR. Both patients not only experienced relief from their primary symptoms, but also reported improvements in overall satisfaction and quality of life. In Case 1, the patient’s persistent throat discomfort improved, along with a significant reduction in her excessive focus on the symptom in daily life, while in Case 2, the significant reduction in pain enabled her to better manage stress and improve daily functioning.

### 4.1 The overlap between SSD and OCSD: therapeutic implications of VOR’s multimodal mechanism

As a multimodal antidepressant, VOR acts not only on 5-HT transporter but also on multiple 5-HT receptors, including agonism at the 5-HT1A receptor, antagonism at the 5-HT3 and 5-HT7 receptors, and partial agonism (functional antagonism) at the 5-HT1B/1D receptors. VOR is thought to contribute to antidepressant and anxiolytic effects by inhibiting local negative feedback in the dorsal raphe nucleus (4), disinhibiting pyramidal neurons by suppressing GABAergic interneurons in the prefrontal cortex and hippocampus (5), and promoting presynaptic release of arousal-promoting neurotransmitters such as norepinephrine, dopamine (DA), acetylcholine, and histamine (6). While these mechanisms suggest potential broad antidepressant and anxiolytic effects, clinical evidence supporting its anxiolytic efficacy is limited. Some small-sample studies and case reports have reported effectiveness in treating patients with major depressive disorder patients with comorbid social anxiety disorder (10) or with a history of trauma (11), as well as preventing panic attacks in patients with panic disorder (12). However, a meta-analysis has shown no significant efficacy in treating generalized anxiety disorder (13, 14). This evidence indicates that the efficacy of VOR for anxiety is not strong. This discrepancy between receptor profiles and clinical outcomes suggests that the mechanisms of action may not fully translate into consistent clinical benefits for anxiety.

Meanwhile, the heterogeneous nature of anxiety and stress-related disorders is increasingly recognized. Specifically, OCD and its spectrum disorders are distinct from other anxiety disorders. OCD was categorized under anxiety disorders in DSM-IV-TR (15), but this heterogeneity led to the creation of a new category of “obsessive-compulsive and related disorders (OCRD)” separated from anxiety disorders in DSM-5 (8, 16). This new category is often referred to as OCSD, reflecting the broader range of related conditions (17, 18).

Additionally, some indirect evidence suggests a potential connection between SSD and OCSD. Hypochondriasis (HC) is often considered part of the OCSD due to its preoccupation with illness resembling obsessive thoughts, and HC shows a high rate of comorbidity and familial occurrence in OCD patients (19–21). Similarly, SSD involves an excessive focus on health concerns (22, 23), which is similar to HC. Given the relationship between HC, SSD, and OCD, HC is classified as obsessive-compulsive or related disorders in the ICD-11 (24), while it is mostly categorized as SSD in the DSM-5 (8, 22). One cognitive mechanism that further links SSD and OCSD is somatosensory amplification, where normal bodily sensations are perceived as intense and distressing (25). This heightened perception of somatic sensations is a hallmark of SSD (26) and is closely tied to emotional factors, particularly catastrophizing, which involves rumination, magnification, and feelings of helplessness, are thought to play a critical role in this process (26–28). Notably, rumination, characterized by persistent focus on distressing thoughts or sensations, has been shown to correlate with the severity of OCD symptoms (29), further suggesting shared cognitive pathways between SSD and OCSD.

Neurobiologically, regions such as the amygdala, insular cortex, and anterior cingulate cortex are broadly implicated in anxiety disorders (30, 31), whereas areas including the orbitofrontal cortex (OFC) and ventromedial prefrontal cortex—which are adjacent and partially overlapping (32)—have been suggested as more specifically involved in OCD (33, 34) and stress-related somatic symptoms (35). However, it should be noted that the stress-related somatic symptoms include not only SSD, but also fibromyalgia, psychosomatic disorders, and subthreshold states. Furthermore, although current evidence is limited to case reports, the observed augmenting effects of antipsychotics on SRIs in treating SSD (36–39) suggest that the neuropathology of SSD may overlap with that of OCD. These findings point to potential shared neural circuits, supporting the hypothesis that SSD may be considered part of the OCSD. Further studies, including randomized controlled trials, are necessary to confirm this relationship and clarify the underlying mechanisms.

In light of the relationship between SSD and OCSD discussed above, we now turn to the consideration of pharmacological treatments for SSD. SRIs are effective in both OCD and other anxiety disorders, but what distinguishes OCD is the involvement of the mesolimbic DA pathway and the effectiveness of D2 antagonists (20, 40). While D2 antagonism are beneficial in treating OCD patients (41), they carry risks of side effects such as extrapyramidal symptoms, hyperprolactinemia, and neuroleptic malignant syndrome. Recently, the 5-HT3 receptor has emerged as another promising target. Meta-analyses have shown that 5-HT3 antagonists are effective for treating OCD, both as monotherapy (42) and as augmentations to SRIs (42, 43). By selectively inhibiting phasic DA release in the mesolimbic pathway, 5-HT3 antagonists may help reduce the side effects often associated with D2 antagonists (3). Additionally, animal and clinical studies also suggest that presynaptic 5-HT1B/1D antagonism promotes 5-HT release in the OFC, potentially contributing to symptom improvement (34). Consistent with these mechanisms, VOR, a 5-HT3 and 5-HT1B/1D antagonist that does not act on D2 receptor, has been shown to be effective in treating OCD patients (44–47).

In the present cases, although neither patient exhibited the obsessive thoughts or compulsive behaviors required for an OCD diagnosis, Case 1’s preoccupation with the unpleasant throat sensation, accompanied by repetitive coughing, aimed at relieving this sensation, and Case 2’s preoccupation with pain, along with overly detailed complaints in the internal medicine setting suggested some OCD-like features and somatosensory amplification. The treatment with VOR led to an improvement in the somatic symptoms of these cases, together with the alleviation of their preoccupation with them. The efficacy of VOR in treating SSD may be attributed to its augmenting effects via 5-HT3 and 5-HT1B/1D receptor antagonism, in addition to 5-HT reuptake inhibition, supporting the hypothesis that SSD may share several neuropathological features with OCD/OCSD.

### 4.2 Other potential therapeutic mechanisms

Additionally, somatic symptoms could also be influenced by the involvement of hypothalamic–pituitary–adrenal (HPA) axis and inflammatory pathways. Numerous studies have shown the association of HPA axis and inflammation with fibromyalgia (48, 49), psychosomatic disorders (50, 51), and conversion disorder/functional neurological symptom disorder (52, 53), all of which should be differentiated from SSD. Whether inflammation is elevated in SSD remains a topic of debate, but recent reports from two groups suggest the involvement of inflammatory pathways in SSD (54) and somatic symptom and related disorders (SSRD) (55). It is important to note, however, that neither group was able to fully explain the mechanism by which inflammation leads to the development of SSD or SSRD, and the causal relationship between physical symptoms and the inflammatory process remains unclear, so it is possible that inflammation was a stress response caused by SSD or SSRD.

Given the suspected role of inflammation in SSD, it is relevant to examine the anti-inflammatory properties of antidepressants, particularly VOR. Antidepressants are known to affect the HPA axis and inflammatory pathways. For instance, SSRIs have been shown to prevent increases in inflammatory cytokines such as interleukin (IL)-1β, IL-6, tumor necrosis factor-α, and interferon-γ (56), as well as reduce quinolinic acid synthesis (57). Some SSRIs have also been shown to exert effects on the HPA axis (58), they have been shown to exert effects on this system. Furthermore, VOR has been found to promote anti-inflammation effects in macrophages (59), increase the anti-inflammatory cytokine IL-4 (56), and inhibit cyclooxygenase-1/2 (60), suggesting a stronger anti-inflammatory effect than SSRIs. Moreover, as a treatment for post-COVID-19 condition, VOR has shown significant cognitive improvement in patients with elevated CRP levels (61), which further supports VOR’s anti-inflammatory effects. These findings suggest that VOR’s anti-inflammatory properties might have contributed to the improvement observed in both cases.

In addition to its anti-inflammatory effects, VOR’s analgesic potential also warrants attention, especially considering the primary symptom in Case 2 was pain. Pain at the spinal level is generally suppressed through the descending inhibitory pathway. 5-HT3 antagonism acts on spinal-level analgesia, while 5-HT7 antagonism promotes pain. Additionally, VOR has a higher receptor affinity for 5-HT3 than for 5-HT7, suggesting that VOR may reduce “true” pain. There have also been reports of VOR’s effectiveness for various types of pain, including chronic pain (62) and neuropathic pain (63) associated with depression, and burning mouth syndrome (64). However, a previous study suggests that spinal 5-HT3 receptor expression increases under sub-chronic stress, leading to heightened physical pain, and that 5-HT3 antagonists can alleviate this “true” pain. Furthermore, as discussed in Section 4.1, psychological factors such as catastrophizing (26) and rumination, which is linked to OCD (29), are involved in somatosensory amplification (25, 27, 28). Serotonergic dysfunction has also been suggested to play a role in this amplification (65). These observations suggest that the boundary between psychological and “true” physical pain might be more ambiguous than expected, and 5-HT3 antagonism might be broadly effective for both psychological and “true” physical pain.

However, evidence supporting the relationship between inflammation and SSD is currently limited, and given the shifting location of pain, Case 2 is more likely associated with psychiatric symptoms than with ‘true’ physical pain. Further research is needed to explore the impact of inflammation and pain mechanisms in SSD.

### 4.3 Limitations

We used the Japanese version of PHQ-15 (9) to assess the somatic symptoms of the cases, because no other suitable scale is available in Japan. Although the PHQ-15 scores were relatively low (2 points for Case 1 and 8 points for Case 2), the patients experienced significant distress. This may be because the PHQ-15, commonly used to assess SSD, quantifies the number of somatic symptoms and their severity on a scale of 0 to 2, making the score more sensitive to the number of somatic symptoms rather than their intensity. These cases highlight the need for the development of a Japanese version of tools like the SSD-12 (66), an SSD scale based on DSM-5 criteria B, which can evaluate the psychological symptoms of SSD (i.e. abnormal cognitive, affective, and behavioral state), irrespective of the number of somatic symptoms. Additionally, we did not administer psychological scales for anxiety and depression. Although the levels of anxiety and depression did not meet the DSM-5 diagnostic threshold, not using these scales prevented us from observing subclinical fluctuations in anxiety or depression. While we primarily focused on SSD core symptoms, especially obsessive-compulsive preoccupation and rumination, which are related to somatosensory amplification, it is possible that the observed improvement was mediated by effects on anxiety or depression. Future research should compare the temporal changes in psychological scales for anxiety and depression with those for core symptoms of SSD.

Additionally, in Case 2, VOR was chosen over SSRIs due to the patient’s concern about nausea, a common side effect of SSRIs, with the expectation that the antiemetic effect of the 5-HT3 antagonism (67) in VOR would alleviate this concern. However, no clinical evidence currently supports the notion that VOR induces less nausea compared to SSRIs (68, 69). In fact, the patient’s nausea worsened as a side effect of VOR. Therefore, in line with clinical evidence, no advantage of VOR in terms of reducing nausea as a side effect was observed in this case. Furthermore, the lack of direct comparison between VOR and SSRIs in Case 2 limits our ability to assess the superiority or specific benefits of VOR’s effects on 5-HT receptors over other treatments.

Moreover, this study has limitations inherent to the study design of case reports, along with specific factors related to the presented cases. First, the small sample size of only two cases limits the generalizability of our findings. For example, Case 1 involved an elderly patient whose main symptom was throat discomfort, whereas Case 2 involved a young adult whose main symptom was pain due to workplace stress. Additionally, in both cases, the types of physical symptoms were relatively limited. These factors may not represent the full spectrum of individuals with SSD. Second, potential biases such as selection bias, reporting bias, and observer bias may have influenced the results. The cases were not randomly selected, and both patients and clinicians might have had expectations that affected symptom reporting and assessment. Third, the absence of a control group and lack of randomization prevent us from establishing causality between VOR treatment and symptom improvement; without a control group receiving placebo or standard treatment, we cannot rule out the influence of placebo effects, natural fluctuations in symptom severity, or spontaneous remission. Although SSD is a chronic disorder, the follow-up period in our study was relatively short—4 months in Case 1 and 6 months in Case 2—which increases the possibility of confounding factors affecting the results. Given these limitations, future robust studies are needed, including randomized controlled trials with larger and more diverse sample sizes, appropriate control groups using placebo or SSRIs, and extended follow-up periods. Such studies would help address potential biases, account for placebo effects, and consider the influence of psychosocial factors, thereby providing more definitive conclusions about the role of VOR in managing SSD.

## 5 Conclusion

The modulatory effects of VOR on 5-HT receptors, particularly its 5-HT3 antagonistic action, have been shown to be effective for a wide range of psychiatric disorders and symptoms, including depression, anxiety, and pain. Notably, 5-HT3 antagonism suppresses mesolimbic DA, and its effectiveness in treating OCD has been recently reported. In the cases presented, VOR also demonstrated significant efficacy in treating SSD. These cases suggest VOR’s therapeutic potential for SSD and further support the close relationship between SSD and OCD/OCSD.

## Data Availability

The original contributions presented in the study are included in the article/**Supplementary Material**. Further inquiries can be directed to the corresponding authors.
