# Supplementary material for: Vortioxetine as an alternative treatment for somatic symptom disorder: case report
Source: Front Psychiatry. 2024 Nov 7;15:1496072. doi: 10.3389/fpsyt.2024.1496072 (PMC11578982; doi:10.3389/fpsyt.2024.1496072)
Supplement: Supplementary file 1 [file Table1.docx]

Supplementary Material

# Use of Generative AI in Manuscript Editing Including Input Prompts

In compliance with the journal's guidelines, the authors acknowledge the use of ChatGPT (version 4.0, OpenAI) to assist in the editing process of the manuscript. The generative AI was used to improve the clarity, structure, and readability of the text. The authors take full responsibility for the final content, ensuring it was reviewed for factual accuracy and alignment with scientific standards.

The following are the initial and final prompts used during the editing process:

Initial Prompt:

"I am writing a case report on the effectiveness of vortioxetine for SSD. The structure of the Discussion section will proceed as follows: 4.1 Broad Antidepressant and Anxiolytic Effects of VOR, 4.2 Shared Therapeutic Mechanisms Between SSD and OCSD, 4.3 Other Potential Therapeutic Mechanisms, and 4.4 Limitations."

Final Prompt:

"Are there any points of concern in the overall discussion?"

No content generated by the AI was included without thorough review and revision by the authors.
